# HIV status disclosure and associated outcomes among pregnant women enrolled in antiretroviral therapy in Uganda: a mixed methods study

**DOI:** 10.1186/s12978-017-0367-5

**Published:** 2017-08-30

**Authors:** Rose Naigino, Fredrick Makumbi, Aggrey Mukose, Esther Buregyeya, Jim Arinaitwe, Joshua Musinguzi, Rhoda K. Wanyenze

**Affiliations:** 1 GF-PMTCT Study, Makerere University School of Public Health, Kampala, Uganda; 2 Department of Epidemiology and Biostatistics, Makerere University School of Public Health, Kampala, Uganda; 3 Department of Disease Control and Environmental Health, Makerere University School of Public Health, Kampala, Uganda; 4 Ministry of Health, Kampala, Uganda

**Keywords:** HIV status disclosure, Spousal support, Stigma, Discrimination, Violence

## Abstract

**Background:**

Disclosure of HIV positive status to sexual partners is promoted by HIV prevention programs including those targeting the prevention of mother-to-child transmission. Among other benefits, disclosure may enhance spousal support and reduce stigma, violence and discrimination. HIV status disclosure and associated outcomes were assessed among a cohort of women, newly initiating lifelong antiretroviral therapy in Uganda between October 2013 and May 2014.

**Methods:**

This was a mixed method study, drawing data from a prospective cohort study of 507 HIV positive pregnant women on lifelong antiretroviral therapy, who were followed for four months to determine disclosure and its outcomes. Women were recruited from three facilities for the cohort study; in addition, fifty-seven women were recruited to participate in qualitative interviews from six facilities. Factors associated with spousal support and negative outcomes were determined using random-effects logistic regression in two separate models, with prevalence ratio as measure of association. In-depth interviews were used to document experiences with disclosure of HIV status.

**Results:**

Overall HIV status disclosure to at least one person was high [(375/507), 83.7%]. Nearly three-quarters [(285/389), 73.3%], had disclosed to their spouse by the fourth month of follow up post-enrolment. Among married women, spousal support was high at the first 330/407 (81.1%) and second follow-up 320/389 (82.2%). The majority of women who reported spousal support for either antenatal care or HIV-related care services had disclosed their HIV status to their spouses (adj.PR = 1.17; 95% CI: 1.02–1.34). However, no significant differences were observed in the proportion of self-reported negative outcomes by HIV status disclosure (adj.PR = 0.89; 95% CI: 0.56–1.42). Qualitative findings highlighted stigma and fear of negative outcomes as the major barriers to disclosure.

**Conclusion:**

HIV status disclosure to partners by pregnant women on lifelong antiretroviral therapy was associated with increased spousal support, but was impeded by fear of adverse outcomes such as stigma, discrimination and violence. Interventions to reduce negative outcomes could enhance HIV status disclosure.

## Plain English summary

Disclosure of HIV positive status is a phenomenon that has not been fully explored with the scale up of lifelong HIV treatment for pregnant women. This study examined 507 pregnant women newly diagnosed with HIV who began treatment in Uganda and were followed for four months. The aim of this study was to determine disclosure of HIV positive status and associated outcomes, as well to explore experiences with HIV status disclosure by in-depth interviews with 57 women.

Overall, nearly three-quarters (73.3%) disclosed their HIV status to their spouses by the fourth month. Other persons most commonly disclosed to were mothers and sisters. Four-in-five women (81.1%) who were married at the time of the interview received spousal support. Barriers to disclosure of HIV status included stigma and the fear of negative outcomes.

HIV positive status disclosure was associated with spousal support but remains a challenge among women living with HIV for fear of negative outcomes. Prevention of mother-to-child transmission programs in the context of high levels of stigma, discrimination and violence should integrate interventions to reduce negative outcomes.

## Background

Despite the considerable efforts made in recent decades to improve access to antiretroviral regimens, HIV status disclosure remains central to improving both maternal and child health outcomes [1]. In Uganda, out of a total of 1,493,164 pregnant women tested during antenatal care (ANC) in 2014, 8% (122,753) were found to be HIV positive [2].

In an effort to eliminate mother-to-child transmission of HIV, antiretroviral regimens have changed over time [3]. As a result, Uganda’s prevention of mother-to-child transmission (PMTCT) policy was revised to incorporate option B+ in October 2012, following efficacy studies in Malawi and Zambia [4, 5]. Option B+ involves use of lifelong antiretroviral therapy (ART) for HIV positive pregnant and lactating women irrespective of the CD4+ cell count. The proportion of HIV positive mothers who received antiretroviral drugs (ARVs) for elimination of mother-to-child-transmission (eMTCT) increased to 84.0% in 2014 [6]. However, access to ARVs in pregnancy is still below the targeted eMTCT goal of 90% [7]. As more HIV-infected pregnant women receive care with new treatment strategies, such as Option B+, programs will need to ensure retention in services. Improved retention will result in benefits, such as, decreased resistance to ARVs and increased survival for the mother-baby pair [6–8].

Previous studies have demonstrated non-disclosure of HIV positive status and stigma as factors associated with poor retention in care [9]. A study done in Kenya, showed that pregnant women living with HIV who had not disclosed their status had the lowest levels of PMTCT service utilization; 21% of women who had not disclosed gave birth in a health facility compared to 49% who had disclosed [1]. Previous studies have shown that most women disclose their HIV status prior to childbirth and their decisions to disclose are influenced by levels of perceived stigma [10]. Persons living with HIV (PLHIV) are often encouraged to disclose their HIV status so as to enhance social supports [11]. Although researchers have argued that lifelong ART may reduce stigmatization of pregnant and lactating women [12], there is limited evidence of their experiences of disclosure including the positive and negative outcomes in the context of lifelong ART. HIV status disclosure and its outcomes were assessed at the second and fourth month post-enrollment among a cohort of pregnant women newly initiating lifelong ART.

## Methods

### Study sites

The study was conducted at six health facilities located in the three districts, Mityana, Masaka and Luwero, in central Uganda. These facilities were among the first to provide lifelong ART in October 2012. These facilities were selected because they had fully operational option B+ programs. Inclusion of both hospitals and lower level facilities (III and IV) provided varying experiences and models of PMTCT.

#### Models of PMTCT services

In Masaka hospital, ANC attendees eligible for lifelong ART were given pre-ART education but ART initiation was deferred for two weeks if the woman was not ready to initiate treatment on the same-day. However, in Mityana hospital and Luwero HC/IV, ART eligible women were immediately initiated on ART and were offered continuous counselling through family support groups. The family support groups (FSGs) were composed of HIV positive pregnant and breastfeeding women as well as their spouses. The groups met once every month at the health facility to receive education and share their experiences.

### Study design

Quantitative data were obtained from a prospective cohort of 507 HIV positive pregnant women attending ANC services. Women were offered lifelong ART during pregnancy as per the Option B+ guidelines, however, some were deferred when not ready. Women initiated on lifelong ART were followed up at two and four months after enrolment to determine HIV status disclosure and associated outcomes. In-depth interviews (IDI) with 57 purposively selected HIV positive women were conducted between February and May 2014 to document their journey following initiation of ART.

### Eligibility

HIV positive pregnant women attending ANC at the designated facilities, who were either ART naïve or recently initiated (within 4 weeks) ART, were eligible for the study.

### Sample size

A total of 500 HIV positive pregnant women were estimated to be needed to address the primary objective of determining the prevalence of HIV status disclosure and its outcomes for pregnant women newly initiating lifelong antiretroviral therapy. The sample size was determined based on the following assumptions: percent of HIV+ women enrolled for PMTCT was *p* = 80%, a 5% level of precision, type-I error rate of 5%, a two-sided α = 0.05, a design effect of 2 to account for the within facility clustering, resulting in 492 which was rounded off to 500. Due to concurrent enrolment at all the 3 sites, a total of 507 were recruited.

### Sampling procedure

The attending ANC nurses referred HIV positive pregnant women for screening and enrolment. Enrolment was done consecutively until the required sample size was achieved.

### Data collection methods

All eligible women provided written informed consent before administration of the baseline interview. A structured questionnaire with closed- and open-ended questions was administered. Women were scheduled for follow-up interviews at two and/or four months after enrolment, to coincide with their next planned ANC or ART visit for convenience. All questionnaires were administered by well-trained and experienced study interviewers stationed at the study facilities.

In-depth interviews (IDI) were conducted with women in three categories: i) good ART adherers, *women who were consistently adherent to ARV refill schedules and drugs based on the clinic records*; ii) poor ART adherers, *women who were known to have missed drug refills and to be struggling with taking their medication* and iii) delayed ART acceptors, *women who did not start treatment for over four months*. Respondents were purposively selected based on client history of ART treatment for option B+. An interviewer experienced in qualitative data collection conducted all IDIs. The IDI guide covered socio-demographic characteristics as well as themes on women’s experiences dealing with HIV status disclosure and outcomes. All interviews were audio recorded.

### Data collection tools

The data collection tools included an eligibility screening form, baseline and follow-up questionnaires. All data collection tools were translated into Luganda, the commonly spoken native language.

### Measures

Data collected included socio-demographic characteristics, history of HIV-testing, HIV disclosure status, and outcomes of disclosure which included HIV-related stigma, discrimination, violence and spousal support to attend ANC, postnatal care and adherence to ART treatment. The primary outcome variable was ‘*HIV positive status disclosure’* while the secondary outcome variables were ‘*outcomes or consequences of disclosure’*.

### Quantitative data management

All questionnaires were reviewed, any edits were done immediately on site with approval from the study coordinator. Each questionnaire was independently entered twice using CSPro version 5. The data manager compared both data entries to identify and correct any inconsistencies.

### Statistical analysis

Descriptive analyses were conducted to generate statistics on women’s characteristics. The prevalence of disclosure was determined as the number of women reporting disclosure to anyone at each visit and cumulatively. The percent of women reporting spousal support including money, escorting or reminders of ANC/PNC or ART services were determined, as well as self-reported experiences with perceived stigma, discrimination or violence.

Factors associated with i) spousal support and ii) HIV-related adverse outcomes (stigma, discrimination or violence) as primary outcomes were determined, where HIV+ status disclosure was the primary explanatory variable and prevalence ratio (PR) as the measure of association. All factors significant at *p* < 0.15 in the bivariate analysis or potential risk factors or already known covariates from previous studies were included in the multivariable regression model. The generalized linear regression models with Poisson family and log link were used to obtain the PR with their corresponding 95% confidence intervals (CI), adjusting for clustering at the individual level and robust standard error. All statistical analyses were conducted using STATA version 12.

### Qualitative data analysis

For the qualitative data, narratives were transcribed and coded independently by two researchers using Atlas ti version 7.0. Thematic analysis was done based on the predetermined themes of stigma, disclosure, positive and negative outcomes of disclosure. The authors met to agree on the suggested codes.

## Results

### Socio-demographic and clinical characteristics

A total of 925 women were screened for eligibility, and 507 consented and were enrolled; Mityana hospital (*n* = 213, 42.0%), Masaka hospital (*n* = 184, 36.0%) and Luwero HC/IV (*n* = 110, 22.0%). The mean age of women was 25.1 years (SD = 6.2), just over half (51.3%, 262) had completed primary school, and 406 (80.0%) were married. The majority (82.5%) tested and learnt of their HIV positive results for the first time on the day of study enrolment. Nearly half of the women (*n* = 195, 47.5%) reported that their partners had been tested for HIV. Women reported that 42.3% and 46.7% of partners as HIV-positive at the two and four-month follow-ups, respectively. The 57 IDI respondents included 29 (50.9%) poor ART adherers, 21 (36.8%) good ART adherers and 7 (12.3%) delayed ART acceptors. The majority of those interviewed were young mothers aged 21–25, the youngest of whom was 16 and the oldest 42. More than half (*n* = 34, 59.0%) of these women had completed primary education. Close to three quarters (*n* = 41, 71%) of the IDI respondents were married and had at least one previous pregnancy (*n* = 54) (Table 1).

**Table 1:** Enrolment characteristics of women enrolled in option B+

Characteristic	Frequencies	Proportions (%)
Overall	507	100
Mityana hospital	214	42.1
Masaka hospital	183	36.2
Luwero H/C IV	110	21.7
Age (years)*		
Mean(SD)	25.1 (6.2)	
15–24	265	52.2
25–29	145	28.7
30–44	97	19.2
Education level		
None or primary only	262	51.5
Lower secondary (grade 1–4)	209	41.4
Advanced secondary (grade 5–6) + vocational training	32	6.3
University	4	0.8
Marital status		
Never married	61	12.1
Married	406	80.0
Widowed/separated	40	7.9
Occupation		
Agriculture	96	18.9
Salaried	29	5.7
Business/commercial	171	33.6
Casual worker	21	4.2
Not employed	43	8.5
Housewife	117	23.1
Other	30	5.9
Currently taking ART		
Yes (Initiated ART within past four weeks)	138	27.3
No (ART naive)	369	72.7
Ever experienced HIV-related stigma, discrimination or violence		
Yes	45	8.9
No	462	91.1

*Age categories were based on definition of young women (up to 24 years) and fertility across age groups (based on Uganda UDHS data)

### Disclosure of HIV status

Overall, three quarters (*n* = 353, 74.5%) of women reported disclosing their HIV status to at least one person at the first follow-up (FUP1, two months post-enrolment) and 375 (83.3%) had disclosed at the second follow-up (FUP2, four months post-enrolment) (Table 2). Disclosure at two and four months was highest in Masaka hospital (82.0% and 88.8%), followed by Mityana hospital (79.7% and 83.9%) and lowest at Luwero HC/IV (64.0% and 77.0%) (Table 2). At both month two and four, women most commonly disclosed to their spouses [353 (59.5%), 374 (58.0%)], followed by sisters [353 (36.8%), 373 (42.1%)] and mothers [302 (37.7%), 330 (39.4%)]. At visit two (four months post-enrolment) only 11 (2.9%) had not disclosed, 187 (49.6%) disclosed to one person, while 179 (47.5%) disclosed to at least two persons (Table 2). Among non-disclosing women, the reasons included fear of breach of confidentiality (*n* = 49, 72.0%), feelings of shame (*n* = 46, 67.6%), and fears of violence or divorce (*n* = 17, 25.0%). One in ten women who did not disclose (*n* = 7, 10.3%) viewed their HIV positive status as a secret, eleven women (16.2%) described the need for more time to prepare to disclose, and two women thought they would lose financial or social support from their spouses if they disclosed.

**Table 2:** HIV status disclosure, partner HIV testing and stigma at month 2 and 4 follow up visits

Characteristic	Follow up	
	At month 2 post-enrolment Frequencies (%)	At month 4 post-enrolment Frequencies (%)
Partner testing		
Overall	407 (100)	389 (100)
Yes	195 (47.9)	184 (47.3)
No	77 (18.9)	93 (23.9)
Don’t know	135 (33.1)	112 (28.8)
How partner tested		
Tested together during ANC	27 (12.0)	31 (19.1)
Tested together outside ANC	34 (10.0)	19 (11.7)
Tested individually	118 (55.0)	112 (69.1)
Partner HIV status		
HIV negative	104 (53.6)	86 (46.7)
HIV positive	82 (42.3)	86 (46.7)
Don’t know	8 (4.1)	12 (6.5)
HIV status disclosed		
Yes	353 (74.5)	375 (83.7)
Disclosure by study site		
Mityana hospital	157 (79.7)	156 (83.9)
Masaka hospital	132 (82.0)	142 (88.8)
Luwero H/C IV	64 (64.0)	77 (77.0)
Persons most commonly disclosed to		
Spouses	353 (59.5)	374 (58.0)
Sisters	353 (36.8)	373 (42.1)
Mothers	302 (37.7)	330 (39.4)
Friends	353 (14.4)	374 (18.7)
Brothers	352 (12.5)	373 (18.0)
Fathers	269 (9.3)	286 (11.9)
Number of people disclosed to		
0	14 (3.9)	11 (2.9)
1	194 (54.8)	187 (49.6)
2	88 (24.9)	104 (27.6)
3	36 (10.2)	36 (9.6)
4	17 (4.8)	25 (6.6)
5	4 (1.1)	12 (3.2)
6	1 (0.3)	2 (0.5)
Self-reported adverse events		
HIV-related stigma	35 (7.6)	49 (10.8)
Discrimination	25 (5.4)	24 (5.3)
Violence	18 (3.9)	17 (3.7)

### HIV status disclosure and spousal support

About eight in ten women with partners, reported spousal support irrespective of disclosure status: FUP1 [*n* = 330, (81.1%)] and FUP2 [*n* = 320, (82.2%)]. Monetary support to attend ANC was the most commonly reported form of partner support at FUP1 [*n* = 219 (82.9%)] and FUP2 [*n* = 155 (55.3%)] (Table 3). Other forms of spousal support included money to facilitate transport to the health facility to retrieve their medications [*n* = 154 (58.1%), *n* = 158 (55.4%)], reminders to attend ANC [*n* = 176 (66.7%), *n* = 137 (48.9%)] and being accompanied to ANC visits [*n* = 23 (8.7%), *n* = 28 (10.0%)]. Spousal support for adherence to ART was present among many who disclosed their status. This type of support included being accompanied to retrieve their medications [*n* = 19 (8.4%), *n* = 14 (10.6%)] and reminders to take their medication on time [*n* = 226 (85.3%), *n* = 132 (46.3%)] (Table 3). Spousal support was higher among women who had disclosed to their spouses [*n* = 265 (80.3%) at FUP1 *n* = 285 (89.0%) at FUP2] than those who had not disclosed [*n* = 65 (19.6%) at FUP1 and *n* = 35 (10.9%) at FUP2] (Table 3). Spousal support included support for non-HIV specific services, for instance being accompanied to antenatal or postnatal care was not necessarily based on disclosure status.

**Table 3:** Type of spousal support received by women disclosure status

	Follow up visit 1 (2 months post-enrolment)		Follow up visit 2 (4 months post-enrolment)	
	Disclosed *(n = 265)*	Not Disclosed *(n = 65)*	Disclosed *(n = 285)*	Not Disclosed *(n = 35)*
Type of support, %				
Gives me money to attend antenatal care	82.9	81.5	55.3	64.7
Reminds me to attend antenatal care	66.7	55.4	48.9	47.0
Escorts me to antenatal or postnatal care	8.7	4.6	10.0	0
Reminds me to take ART	85.3	0	46.3	0
Gives me money to pick-up ART	58.1	0	55.4	0
Escorts me to refill ART	8.4	0	10.6	0

Results include only the 407 women at FUP1 and 389 at FUP2 who had sexual partners at the time of interview

In the multivariable regression model, HIV status disclosure to a spouse was associated with 17% higher prevalence of spousal support (adj.PRR = 1.17; 95%CI: 1.02–1.34) (Table 4). Spousal support was 17% higher for women with advanced education or vocational training compared to women with primary or no education [adj.PRR = 1.17(1.05, 1.31]. Spousal support varied between recruitment sites. Compared to Mityana facility, women in Masaka had a 10% higher spousal support (adj.PRR = 1.10; 95%CI: 1.02–1.17) while women in Luwero had a 23% lower spousal support (adj.PRR = 0.77; 95%CI: 0.66–0.90) (Table 4). The association between spousal support and HIV status disclosure to spouse was adjusted for age, level of education, marital status, employment, partner testing, type of visit (antenatal versus postnatal visit), health facility and alcohol/drug use.

**Table 4:** Risk-adjusted analysis for the association between spousal support and status disclosure to sexual partner

Variable Name	Adjusted Analysis	
HIV status disclosure to sexual partner*	PRR^a^	95%CI
Not disclosed to sexual partner	1.0	
Disclosed to sexual partner	*1.17*	*1.02, 1.34* **
Age category		
15–24	1.0	
25–29	0.99	0.91, 1.08
30–44	1.00	0.91, 1.10
Marital status		
Never married	1.0	
Married	1.11	0.96, 1.28
Widowed/separated	0.79	0.59, 1.05
Occupation		
Unemployed	1	
Homemaker	0.93	0.84, 1.03
Employed	1.00	0.92, 1.08
Education level		
None or primary only	1	
Lower (grade 1–4) secondary	0.99	0.91, 1.07
Advanced (grade 5–6) secondary + vocational training	*1.17*	*1.05, 1.31* **
University	0.97	0.69, 1.35
Type of visit		
Antenatal visit	1	
First postnatal visit	0.97	0.91, 1.05
Subsequent postnatal visits	0.89	0.77, 1.03
Partner testing		
No/don’t know	1	
Tested	*1.17*	*1.08, 1.26* **
Enrolment health facility		
Mityana hospital	1	
Luwero H/C IV	*0.77*	*0.66, 0.90* **
Masaka hospital	*1.10*	*1.02, 1.17* **
Alcohol, drug/substance use		
No	1	
Yes	1.08	0.98, 1.20

PRR^a^ means adjusted prevalence risk ratios, *main independent variable, Results include only the 407 women who had sexual partners at the time of interview, **denotes a significant difference at *p* < 0.05

### Women’s experiences disclosing their HIV positive status

Women’s experiences disclosing their HIV positive status varied among good adherers, poor adherers and delayed ART acceptors in terms of how and whom they disclosed to. The process of disclosing for the majority of women interviewed, required time. Most women found it very difficult to break the news to their spouses and tried to go about it by persuading their partners to go for HIV testing*.* However, some women reported that their spouses refused to be tested even after making several attempts to persuade them, which further compelled them to conceal their HIV positive status. Almost all the good ART adherers (*n* = 17, 80.9%) had disclosed their HIV status to their spouses. Disclosure to their spouse was largely facilitated by prior knowledge of their HIV status and a good relationship with their spouses. As demonstrated in the quantitative results, women who disclosed to their spouses reported being supported to adhere to their medication and clinic appointments.

*What has helped me is because my spouse is aware that am sick [meaning HIV positive]. I normally don’t stay with him, but when it comes to 8:00 pm, he calls me [by telephone] saying, are you going to swallow the ARV drug, have you swallowed it? --Good ART Adherer (Luwero HC/IV).*

There were a few cases of good ART adherers (*n* = 5) who described disclosure difficulties. Some women took their medication in hiding. For example, one woman reported keeping her drugs in a teddy bear to keep them hidden.

*In the sitting room, there are many teddy bears, which look like decorations. So I pick my teddy bear as if I am playing with it and go to the store, swallow my drug and come back. --Good ART adherer (Katikamu HC/III).*

In contrast to the good adherers, almost all the poor adherers (*n* = 27) and delayed acceptors (*n* = 30) had not disclosed their HIV status to their spouses for fear of domestic conflict including separation.

*I feared because I did not want the man to see me taking the drugs. He told me that, if they test me for HIV and am found with the HIV virus, we will divorce. --Delayed ART acceptor (Mityana hospital).*

Five of the 29 poor ART adherers who disclosed their status, did so to a female relative particularly their mother, aunt or sister.

*When I went home, I explained to our elder sister and told her that they have told me that I have HIV. She said don’t fear, you have to start taking the drug so that the baby does not get infected. I said okay, I went with my elder sister to the health worker, they counseled me and I started the drug.* --*Poor ART adherer (Luwero HC/IV).*

Non-disclosure to spouses was mainly because of fear of losing support from their partners and fear of being accused of infecting their partner.

*Women see their husbands as their spouse and fathers, they know if they tell their husbands that they are like this [meaning HIV positive], they will be sent away [meaning divorce]. Even if he knows that he is the one who brought that thing [meaning infected his spouse with HIV], he will say that it is the woman who brought it. That’s why the majority of women keep quiet saying that if I tell the man he will chase me away. Who will look after me at home? -- Good ART adherer (Katikamu HC/III).*

Having poor relationships with their spouses was reported as a barrier to disclosure. Women in polygamous relationships experienced difficulties disclosing their HIV status to their husbands for fear of being abandoned for other wives and many women cited fear of violence.

*That man [my husband] beats me. I do not have a good relationship with him.*

*I share my secrets with my mum, she told me not to worry but to continue swallowing my drugs. —Poor ART adherer (Masaka hospital).*

Most of the women interviewed (*n* = 33) mentioned non-disclosure to their spouses as a key barrier to good ART adherence. They experienced disclosure difficulties and consequently struggled to adhere to their treatment. Their challenges ranged from swallowing their drugs in hiding to missing their ART doses and clinic appointments.

*My husband was around. I feared to tell him that I am going to pick the drugs. He doesn’t know that I am taking the drugs. --Poor ART adherer (Katikamu HCIII).*

*I target when he has gone to bathe, after preparing water for him, that’s when I swallow it. By the time he returns from outside [bathroom], I would have already finished swallowing. --Poor ART adherer (Katikamu HCIII).*

*If he doesn’t move away; I get it [meaning the ARV drug] and go outside the house, remove it and tell the child to get me water and I swallow it…. For a long period, I have been swallowing it but he doesn’t know. --Poor ART adherer (Katikamu HCIII).*

### HIV-related stigma, discrimination or violence

Table 2 shows the self-reported adverse events - stigma, discrimination, violence - at month two and four. The percent of women who reported experiencing stigma [35 (7.6%) vs 49 (10.8%)], discrimination [25 (5.4%) vs 24 (5.3%)] and violence [18 (3.9%) vs 17 (3.7%)] were similar. Prevalence of negative outcomes at FUP1 was highest in Luwero (16.3%), followed by Masaka (14.8%) and Mityana (9.1%) while at FUP2, negative outcomes were most common in Masaka (26.9%) followed by Mityana (8.0%) and least in Luwero (5.2%). Women with no reported negative outcomes indicated the following coping mechanisms; to ignore the actions of those who stigmatize (60.6%), to stay strong and continue taking their medication (22.9%), to seek counsel from health workers (8.3%), to move to communities where their HIV status is not known (6.1%) or to isolate themselves (5.5%).

### HIV status disclosure to at least one person and associated negative outcomes

Table 5 shows the association between the self-reported adverse events (HIV-related stigma, discrimination or violence) and HIV status disclosure adjusting for age, education level, marital status, employment status, type of visit (antenatal or postnatal), enrolment at health facility and alcohol or drug use. In the adjusted analysis, the risk of self-reported adverse events was significantly lower among married women compared to never married women, adj.PR = 0.56; 95% CI: 0.34–0.91, but higher in Masaka compared to Mityana women, adj.PR = 2.25; 95% CI: 1.44–3.52. However, no significant differences were observed by HIV status disclosure adj.PR = 0.89; 95% CI: 0.56–1.42.

**Table 5:** Risk-adjusted analysis for the association between negative outcomes and HIV status disclosure to someone

Variable Name	Adjusted Analysis	
HIV status disclosure to at least someone*	PRR^a^	95%CI
Not disclosed to any one	1.0	
Disclosed to at least someone	0.89	0.56, 1.42
Age category		
15–24	1.0	
25–29	1.13	0.73, 1.74
30–44	1.40	0.86, 2.28
Marital status		
Never married	1.0	
Married	*0.56*	0.34, 0.92**
Widowed/separated	*0.13*	0.03, 0.53**
Occupation		
Unemployed	1.0	
Homemaker	0.82	0.48, 1.41
Business/wage	0.92	0.60, 1.42
Education level		
None or primary only	1.0	
Lower (grade 1–4) secondary	0.85	0.58, 1.27
Advanced (grade 5–6) secondary + vocational training	0.82	0.36, 1.86
University	1.82	0.98, 3.37
Type of visit		
Antenatal	1.0	
First postnatal visit	0.78	0.53, 1.17
Subsequent postnatal visits	1.29	0.77, 2.15
Enrolment health facility		
Mityana hospital	1.0	
Luwero H/C IV	1.16	0.66, 2.02
Masaka hospital	*2.25*	*1.44, 3.52*
Alcohol, drug/substance use		
No	1.0	
Yes	1.55	0.92, 2.59

PRR^a^ means adjusted prevalence risk ratios,

*main independent variable

**denotes a statistically significant difference at *p* < 0.05

### Women’s experiences dealing with HIV-related stigma, discrimination and violence

Women had varying experiences with HIV-related stigma, discrimination and violence. Most women (*n* = 45, 78.9%), both good and poor ART adherers, felt that HIV-related stigma had reduced within their communities. The most commonly reported form of HIV-related stigma was the fear of stigma from others (Table 6). Some women felt shame or fear of being seen using services at the HIV clinic and some missed their clinic appointments as a result.

*When I was given the drug, I said …aha…how will I swallow this? The people I stay with at home have a lot of “lugambo” [gossip]. I don’t even use the container for the drugs. I put the drugs in a polythene bag and throw away the container. --Good ART adherer (Mityana hospital*).

**Table 6:** Variation in the model of care for PMTCT programs by health facility

Health facility	Health care workers involved in PMTCT service delivery	Model of care for initiating Option B+	Strategies for enhancing retention in care
Mityana Hospital	There are 8 health care workers involved in PMTCT: 3 nursing officers, 3 senior enrolled midwives, 2 expert clients and 2 data clerks.	At ANC, mothers are started on ARV on the same day they test positive. Formal postnatal care appointments are scheduled. Mothers are given ARVs monthly and after delivery until the baby is 6 months, thereafter, drug refills are given every 2 months or more based on the mother’s request. After delivery, women who are due for transfer from ANC are escorted by an expert client to the ART clinic and handed over to the provider on duty.	Mildmay Uganda facilitates expert clients to follow up clients who do not return for their ART refills. Mildmay Uganda also supports routine counselling and health education through monthly family support group meetings. Routine outreach activities are conducted.
Luwero HC IV	There are 13 health workers involved in PMTCT: 2 medical officers, 9 registered midwives and 2 nursing assistants.	Mothers are started on ARVs during pregnancy although some who might refuse to start ART are asked to return when they are ready. Formal postnatal appointments are scheduled. Frequency of ART drug refills postpartum is every 2 months for mothers on Option B+ who are compliant and monthly for those who are not compliant.	PREFA facilitates expert clients to follow up clients who do not return for their ART refills. PREFA also supports routine counselling and health education through monthly family support group meetings. Routine outreach activities are conducted.
Masaka RRH	There are 14 health workers involved in PMTCT: 2 nursing officers, 8 midwives, 2 counsellors and 2 expert clients	The decision to start taking ARVs entirely lies with the client. Mothers who are not ready are encouraged to return at a later date to start ARV therapy. All appointments with the mother are tied to those of the baby (mother-baby). EID mother-baby pair reviews are done monthly post-delivery for the first 6 months and continued 3-monthly until the baby is 18 months.	Uganda Cares facilitates expert client to follow up clients who do not return to the facility. No family support group meetings are provided. Routine outreaches activities are conducted.

Some women experienced stigma, and therefore, reported not returning to the clinic, while others opted to move to another facility where they were ‘*not known*’.

*Going back to get other doses is a problem to me because most people know me and I don’t want anybody to see me, because they will go back and tell my parents.* --*Poor ART adherer (Luwero HCIV).*

Others reported the fear of losing social standing within their families or community, which influenced their decision to disclose and subsequently whom they disclosed to.

*They say that the sick (meaning persons living with HIV) should not contest for a political position in society and should not discuss anything in public. They say aah…that one is going to die tomorrow. --Poor ART adherer (Luwero HCIV).*

## Discussion

This study assessed the prevalence of HIV status disclosure, as well as the outcomes for pregnant women newly initiating lifelong ART in Uganda. HIV status disclosure to at least one person was high, and increased to over 80% after four months. Disclosure to a spouse was associated with increased support, including support for regular clinic attendance and ART adherence. Although relatively uncommon, lack of disclosure and fear of stigma were major barriers to ART adherence. Some women did not disclose their status for fear of losing support from their partners. This finding corresponds with a qualitative study in a similar setting where the fear of disclosure among HIV positive women was influenced by their economic dependency on men, among other factors [13]. The biggest barrier to spousal disclosure was the fear of negative outcomes, especially divorce. Disclosure is a process that requires preparedness and time, as described in the process-oriented framework or disclosure processes model [14, 15]. It is thus not surprising that disclosure increased over time [10]. Similar to previous studies done in the general population, women in this study reported disclosing more often to their spouses, mothers, and sisters as opposed to their fathers and brothers [10, 16]. The frequency of disclosure to a spouse surpassed disclosure to mothers and sisters or other family member, and was high when compared to another study in Uganda which showed that 83.8% of women disclosed their HIV status to their partners following antenatal HIV testing [17]. Almost all women who disclosed to their spouses, reported receiving adherence support in the form of transport and reminders to take their ARVs [11, 18]. Spousal support in the form of reminders to take their ARVs on time decreased from 85.3% to 46.3% by the fourth month post-enrolment. Spousal reminders may enhance medication adherence, which may make reminders unnecessary over time as adherence increases. This effect was demonstrated by a meta-analysis which showed an increase in adherence for at least one of the reminder groups compared to the control group receiving no reminder intervention [19]. Spousal support varied between recruitment sites; this may be attributed to their different models of PMTCT service delivery (Table 6). Spousal support was higher among women in Masaka, this may be because Masaka hospital allowed time to decide and prepare prior to starting treatment. Women whose sexual partners had been tested for HIV and those who had completed advanced level education reported greater spousal support. This finding is not surprising, as previous studies indicate that higher education and knowledge of partner’s HIV status enhance disclosure and spousal support [20]. Similarly, having a sexual partner who had been tested makes it easier for women to disclose their HIV status [21]. Interventions to enhance HIV status disclosure and partner support should address women’s fears and support for partner testing. Such interventions may include efforts to scale up family support groups. In this study, women made various attempts to disclose and to encourage their partners for testing, with varying levels of success; this could be a point of intervention for PMTCT services, as disclosure to spouses increases women’s participation in PMTCT programmes [22].

HIV-related stigma and other negative outcomes such as, discrimination and violence were rarely mentioned at baseline, this may be because women had just learned their HIV status. At follow-up visits, experiences of HIV-related stigma, discrimination and violence remained low (3.9%–10.8%). This is similar to rates reported in a systematic review in developing countries, where less than 15% (3.5% -14.6%) of women reported intimate partner violence following disclosure [23]. A cohort study done in Uganda, found that women who reported abuse associated with antiretroviral therapy program participation, also had pre-existing history of domestic violence [24]. Male involvement campaigns may attenuate or avert occurrences of these undesirable outcomes (stigma and violence). In South Africa, an integrated intervention designed to reduce gender-based violence showed reduced violence against women [25]. Another study done in Uganda to assess the impact of a combination of intimate partner violence (IPV) prevention campaigns and HIV services showed reduced self-reports of IPV among women in the male involvement arm [26]. There was a higher risk of HIV-related stigma, discrimination and violence among women who were never married. This higher level of negative outcomes could be attributed to the role of family support group (FSG) meetings which were largely attended by the married (stay home) women. In these FSGs, women regularly met at health facilities to provide mother-to-mother peer support and discuss issues related to stigma and disclosure. The qualitative findings showed that many women, especially the poor adherers were struggling with internalized stigma expressed by feelings of shame and the fear of negative outcomes. Women went to great lengths to hide their ARV drugs from their spouses, some missed their clinic visits while others preferred to obtain drug refills from distant clinics to avoid encountering people they knew [27]. Recent studies in Uganda have shown an increase in internalized stigma among PLHIV presenting for treatment [28], and significantly higher levels of internalized stigma among women [29]. More than half (52.2%) of the women in this cohort were young women (<24 years). Many participants were not employed outside the home and were likely financially dependent on their spouses to maintain their treatment regimen, a situation which increases vulnerability and insecurity [30]. Comprehensive interventions to address internalised stigma should thus be sensitive to underlying social challenges.

Similar to other studies, these findings indicate that many partners tested HIV positive (>40%) [31]. Thus, in addition to improving PMTCT programmes, disclosure and partner testing at ANC, may support getting HIV infected partners into care.

### Study limitations

The present study could not ascertain if the violence and other negative outcomes reported were precipitated by testing and disclosure. Given that the prevalence of such outcomes was higher among those who did not disclose, it is possible that such women may have avoided disclosure due to previous non-HIV related negative experiences. In addition, the male perspective could have helped confirm any tendencies to overestimate disclosure and positive outcomes due to social desirability. Analysis of qualitative data was limited to predetermined themes which made it difficult to explore themes emerging from the data. The quantitative analysis was not sufficient to determine the type of stigma, whether felt or external stigma. The small sample size may limit the findings of this study; however, it provides useful insights into the disclosure experiences and outcomes of pregnant women in Uganda.

## Conclusion

The findings of this study emphasize the major role of HIV status disclosure in enhancing access to services. Perceived stigma was a major barrier to disclosure and to access of PMTCT services. Interventions to reduce stigma, enhance spousal disclosure and testing are needed to maximise the benefits of lifelong ART.

### Recommendations

In the era of lifelong ART for women living with HIV, more research is needed to characterize the perpetuators of HIV-related negative outcomes of HIV status disclosure.

## Abbreviations

ACP: AIDs Control Program
ANC: Antenatal Care
ART: Antiretroviral Therapy
ARVs: Antiretroviral drugs
C I: Confidence Interval
EID: Early Infant Diagnosis
eMTCT: Virtual Elimination of Mother-To-Child-HIV Transmission
FSG: Family Support Group
FUP: Follow Up
GF: Global Fund
H/C: Health Center
HIV: Human Immunodeficiency Virus
MakSPH: Makerere University School of Public Health
MoH: Ministry of Health
MTCT: Mother-to-Child Transmission
PLHIV: People Living with HIV/AIDS
PMTCT: Prevention of Mother-To-Child-Transmission of HIV
PNC: Postnatal Care
PRR: Prevalence Risk Ratio
SD: Standard Deviation
UNAIDS: Joint United Nations Programme on HIV and AIDS
